# Saving Lives: Evaluating Naloxone Distribution in Community Drug and Alcohol Services

**DOI:** 10.1192/bjo.2026.11589

**Published:** 2026-06-30

**Authors:** Anju Vivek Shivram, Frankie Anderson

**Affiliations:** 1 South London And Maudsley NHS Foundation Trust, London, United Kingdom; 2 South West London And St George's Mental Health NHS Trust, London, United Kingdom

## Abstract

**Aims:**

This service evaluation reviewed the distribution of intramuscular and intranasal naloxone between January 2025 and January 2026 within Richmond Community Drug and Alcohol Services, as part of a harm reduction approach for people with opioid use disorder. Naloxone is a well-established opioid antagonist used to reverse opioid-related respiratory depression in emergency situations. The evaluation was particularly relevant to the London Borough of Richmond upon Thames, where drug-related deaths continue to occur despite lower prevalence overall, and where polysubstance use and exposure to potent synthetic opioids increase the risk of overdose among people accessing local services.

**Methods:**

A retrospective review of anonymised naloxone distribution records was undertaken, capturing all kits issued over the 12-month evaluation period. Data included the total number of kits provided and the formulation supplied, either intramuscular or intranasal. Naloxone was offered in line with UK national guidance to people using opioids alone or with others, individuals who inject drugs, those assessed as being at increased risk of overdose, and people likely to witness an overdose. Distribution was supported by brief, practical harm reduction advice. This focused on using low doses and increasing gradually, especially when using a substance for the first time, allowing time for effects to be felt, and recognising that drugs may act at different speeds or vary in strength despite similar appearance. Individuals were also encouraged to inform others when using substances, access buddy or telephone support if using alone, and seek emergency medical help when concerning symptoms occurred. Findings were summarised using descriptive analysis.

**Results:**

During the evaluation period, a total of 237 naloxone kits were distributed. Of these, 75 kits (31.6%) were intramuscular naloxone, and 162 kits (68.4 %) were intranasal naloxone. Intranasal naloxone accounted for the majority of kits provided. This reflects its ease of administration, lower risk of needlestick injury, and suitability for use by people without medical training. Distribution occurred within a population characterised by established opioid dependence, where polysubstance use and exposure to synthetic opioids were an identified risk.

**Conclusion:**

This evaluation supports sustained naloxone distribution as an effective harm reduction measure, with intranasal naloxone facilitating wider access and rapid overdose response. Given its short duration of action, repeat dosing and urgent medical assessment remain essential, particularly with long-acting or synthetic opioids.

